# Energy, protein and iron densities of dabi teff-field pea-based optimised novel complementary flour and its contribution to daily energy and nutrients demand by 6–23-month-old children

**DOI:** 10.1017/S0007114523002581

**Published:** 2024-03-28

**Authors:** Diriba Chewaka Tura, Tefera Belachew, Dessalegn Tamiru, Kalkidan Hassen Abate

**Affiliations:** 1 Department of Nutrition and Dietetics, Institute of Health, Jimma University, Jimma, Ethiopia; 2 Department of Human Nutrition, Wollega University, Nekemte, Ethiopia

**Keywords:** Dabi-teff, Optimised novel complementary food, Energy and nutrient density, Energy nutrient contribution

## Abstract

Inadequate intake of age-specific energy and nutrients is among the prime immediate causes of child malnutrition. Thus, this study aimed to determine the energy, protein and Fe densities of pre-processed dabi teff-field pea-based optimised novel complementary flour and its contribution to daily energy and nutrients demand by 6–8, 9–11 and 12–23 month-old children. The optimal formula at overall optimisation was identified to be 34·66 % dabi teff, 25 % barley, 15 % oats, 15·34 % field pea, 5 % linseed and 5 % maize with response values of 15·74 % protein, 5·09 % fat, 2·26 % ash, 2·88 % fibre, 73·05 % carbohydrate, 1591·72 kJ/100 g (380·43 kcal/100 g) energy, 32·21 mg/100 g Fe, 77·51 mg/100 g Ca and 2·59 mg/100 g Zn. The energy density of the optimised novel complementary flour was 1·27 kcal/g which fulfilled the Pan American Health Organization/WHO recommendation (≥ 0·8 kcal/g), protein density was 4·14 g/100 kcal and the Fe density was 8·47 mg/100 kcal, which was 2·12 to 10·59 times higher than the recommended value where the optimal had demonstrated to contribute more than 100 % of the daily energy and protein demand and notably more than 200 % of daily Fe demand at moderate bioavailability (0·8–4 mg/100 kcal). These findings showed that the daily recommended dietary allowance for energy, protein and Fe could be attained by the developed dabi teff-field pea-based optimised novel complementary flour and its contribution to the children’s daily energy and nutrients demand met the standard, where the product can be used as food-based nutrition intervention to manage protein-energy malnutrition and Fe deficiency anemia in children sustainably.

Adequate nutrition during childhood is a key factor to support the rapid rate of physical growth and mental development that occurs in the first 2 years of life^(1,2)^ as well as for healthy life and future productivity of children^(3,4)^. Stunting or declined linear growth of children usually occurs during the period of complementary feeding, particularly around the end of the second year of life, and ends irreversible^(5)^. Additionally, Fe deficiency anaemia is more prevalent in early childhood (between 6 and 24 months) than at any other time in the life cycle where the major causes are inadequate dietary intake of bioavailable Fe, malaria and parasitic infections^(6,7)^. During this critical time, there is a high dietary requirement of Fe per body weight, where some health firms recommend that infants should be primarily introduced at first to Fe-rich complementary foods such as Fe-fortified or meat foods to meet their Fe requirements and alleviate long-life developmental impairments^(8,9)^.

Infants and young children need adequate energy and a nutrient-dense diet both through exclusive breast-feeding followed by quality complementary feeding^(1,2,10)^. In the first 6 months after birth, breast milk is adequate for energy and nutrient requirements by infants^(2)^ after which the milk is no longer sufficient and children transfer from ideal and uncontaminated breast milk to poor-quality complementary foods (often regular family food) with the highest chance of vulnerability to malnutrition and infectious diseases^(11,12)^.

The energy density of a food refers to the amount of calories per unit weight of the food and is expressed as kcal/g, whereas nutrient density refers to the amount in grams of macro- and micronutrient nutrients in a food per 100 kcal^(13,14)^. The energy needs from complementary foods for infants with ‘average’ breast milk intake in developing countries are approximately 200 kcal per day at 6–8 months of age, 300 kcal per day at 9–11 months of age and 550 kcal per day at 12–23 months of age^(15,16)^. According to FAO/WHO^(12)^ guidelines, the critical values of nutrient contents in formulated complementary food should be 400–425 kcal/100 g energy, ≥ 15 % protein, 10–25 % fat, 64 (sd 4) % carbohydrate, 16 mg/100 g Fe, 500 mg/100 g Ca and 3·2 mg/100 g Zn to be nutritional adequate^(12)^.

Complementary foods need to be far more nutritious compared with family foods to fill the energy and nutrient gap. It should contain protein of high-biological value comprising the essential amino acids. Yet, the opposite is the case in low-income countries. Apart from a major source of essential amino acids, protein is a good energy source at times of energy deprivation^(17,18)^. Low energy intake accompanied by inadequate intake of protein leads to protein-energy malnutrition, the commonest lethal form of malnutrition revealed to us ‘the silent emergency of the world’^(19)^. Thus, infants and young children need adequate protein where the recommended daily amount of protein in grams for a 6–8-month-old child is 9·1 g, for a 9–11-month-old child is 9·6 g and for a 12–23-month-old child is 10·9 g^(15)^. The ratio of energy from protein to the total energy content of a given food, which is referred to us the protein-energy ratio, is one of the indicators used to assess the adequacy and quality of protein in complementary foods. The Codex Alimentarius commission^(12)^ specifies that the daily energy needs from protein (protein-calorie) should be between 6 and 15 % of the total daily energy requirements and typically should not exceed 15 %. This is because while protein is still crucially required for growth and development, a higher protein-calorie ratio beyond 15 % in complementary foods may increase the renal solute load (the non-metabolised nitrogenous end products) and interfere with appetite^(20)^.

In addition to protein, infants and young children require an adequate amount of micronutrients such as Fe, Zn, Ca and vitamins to meet their physiological needs, particularly in the first 2 years of life and they are prone to micronutrient deficiencies and associated diseases^(18,21)^. Ahmed *et al.* ^(22)^ stated that globally, 7·3 % of the disease burden in children is accounted for by micronutrient deficiencies. The contribution of dietary minerals from breast milk declines with a child’s age and would be almost zero after 12 months, which makes these nutrients limiting nutrients^(23)^. The percentage of total daily micronutrient requirement from complementary foods may range from 30 to 100 %. For instance, for 9–11-month-old children, about 97 % of Fe, 86 % of Zn, 72 % of Ca, 76 % of Mg, 81 % of P and 73 % of Na requirements of children 6–24 months should come from complementary food, assuming average breast milk intake^(15,24)^.

Pan American Health Organization (PAHO) and WHO^(15)^ recommend Fe intake by children at 6–8, 9–11 and 12–23 months should be 11, 11 and 7 mg/d, respectively, where these figures are 4·1, 4·3 and 5·8 mg/d for Zn and 400, 400 and 500 mg/d for Ca for the respective age groups. Meeting such high demand is unlikely to be attained through a traditional complementary food, which calls for searching for other promising alternatives such as exploring locally available underutilised/forgotten nutritious cereals and legume crops and developing optimised complementary flour.

The theoretical gastric capacity of infants and young children is 30 g/kg body weight per meal, but their functional gastric capacity is much lower than this figure in usual settings. For instance, Abeshu *et al.* ^(25)^ have reported that the functional gastric capacity of 6–23-month-old children on average was around 20 g/kg body weight per meal for the study done at Wolayita Zone of Ethiopia, whereas Baye *et al.* ^(26)^ had reported a very low functional gastric capacity at 9 g/kg body weight per meal for 12–23-month-old children for the study done at North Wollo of Ethiopia. Because of this lower functional gastric capacity, children need to be fed energy and nutrient-dense food to satisfy their daily demands and served more frequently ^ ^20^^than adults WHO^(20)^. PAHO/WHO^(15)^ recommends the required minimum energy density from processed cereal–legume-based complementary food should be 0·8 kcal/g, protein density should be up to 5·5 g/100 kcal and Fe density needs to be 0·8–4 mg/100 kcal where the required number of meals to be served to children per day was estimated to be 2–3 times for 6–8-month-old children and 3–4 times for 9–23-month-old children to provide adequate daily energy and nutrients requirements by children with additional one or two nutritional snacks based on the appetite of the child.

Application of some simple processing techniques like fermentation, germination, soaking, roasting and dehulling can improve the nutrient densities and absorption of nutrients from cereal–legume foods as well as boost organoleptic qualities^(12)^. Dietary factors such as meat, poultry, fish and citrus fruits are also good sources and important enhancers of non-haem Fe and Zn absorption from plant foods^(27,28)^. But in many developing countries, these products are too expensive and consumed on special occasions only, if not at all, where it is even unthinkable to purchase meat by people with poor socio-economic classes. Currently, the development of energy and nutrient-dense complementary foods from local and readily available nutrient-rich cereals and legumes combinations has received a lot of attention in many developing countries since it is cost-effective, sustainable and can be adapted to different cultural and dietary traditions and locally feasible strategies^(29,30)^. The food-based dietary guidelines by the International Life Science Institute^(31)^ focus on the combination of foods that can meet nutrient requirements rather than on how each specific nutrient is provided in adequate amounts. This guideline was based on the fact that people eat foods, not nutrients or food tablets^(32)^. Recently, nutrition has got an increasing attention with stronger political involvement at both the national and international levels. For instance, the Ethiopian food and nutrition policy and strategy which was recently formulated supports the development and promotion of local production of complementary foods meeting acceptable standards as on one of its operationalising the strategy^(33)^.

Generally, inadequate intake (both in quantity and quality) of age-specific essential nutrients together with infectious diseases is the prime immediate causes of child malnutrition leading to anthropometric deficits (stunting, wasting, underweight), anaemia, impaired cognitive development and finally increased morbidity and mortality. According to the Ethiopian Public Health Institute survey^(34)^, 37 % of children under 5 years old were stunted, 21 % were underweight, 7 % were wasted, 2·9 % were overweight and 57 % anaemic. Thus, it was hypothesised that the development of energy, protein and Fe-dense complementary foods from locally available food crops including the underutilised/forgotten crops that could meet acceptable standards for energy and nutrient requirements by children and its promotion could be used to sustainably manage child malnutrition and its aligned consequences.

The sub-Saharan African region including Ethiopia is endowed with a rich diversity of ‘orphan crops’ having improved nutritional and medicinal values as well as sources of income generation^(35)^. Little or no attention was given to such crops in terms of research and development and policy framework that could promote their extensive agricultural production, industrial utilisation and home consumption. In Ethiopia, the food culture of teff grains, ‘a widely utilised orphan crop’, is both historical, an integral part of the country’s antiquity, and mostly traded domestically where more than 70 % of the Ethiopian population use teff grains as a traditional staple meal.

Dabi teff (*Eragrostis tef*), a farmer variety teff grain, is one of the underutilised/marginalised or forgotten food crops grown in Ethiopia (Oromia region) that could be utilised to combat macro- and micronutrient malnutrition. *Dabi* teff is the ‘afaan oromoo language’ name for an early maturing variety of dark red teff grain. The early maturing property of the grain makes the crop to be harvested twice within one rainy season (at early rainfall called ‘*daabi* gannoo’ and late rainfall called ‘*daabi* birraa’). Farmers in Wollega and Illuababor zones, Western Ethiopia, cultivate *dabi teff* either for its grain seeds or its straw. The contribution of the grains to household food and nutrition security is considerable when viewed through a food security lens while the straw is composed of fine stems used for plastering (finishing) mud hut walls during house construction and rated higher among others for animal fodder especially oxen feeding^(35)^. There are many social beliefs regarding the nutritional and health benefits of *dabi* teff among rural elderly people in particular and consumers in general (Personal communication). Price-wise, the red teff varieties grown in Ethiopia including *dabi* teff are the cheapest. For example, Rashid and Negasa^(36)^ reported that the market price of mixed teff and red teff was 24 and 55 % less than the white teff. In addition to lower prices, red teff varieties have been implicated in the low incidence of anaemia in Ethiopia, which is presumed to be due to the grain’s high Fe content^(37)^. The other cereals and legume components included in our current formulation for optimisation were thoughtfully selected to develop a wholesome and nutritionally complete novel complementary product. For example, the legumes/pulses field pea and linseed were selected because of their naturally containing high amounts of protein and to complement the lysine-limited cereals. Additionally, linseed is a popularly cultivated oil crop by the community in the sample collection area and its oil is among the leading sources of *α*-linolenic acid and *n*-3 PUFA from plant foods^(38)^, which is crucially important for brain neural and cognitive development *in utero* and early childhood^(39)^.

On the other hand, the cereals maize, barley and oats were selected because of their high carbohydrate contents and also for complementing the cysteine, methionine and tryptophan-limited legumes. All these nutritional qualities of the components would make the complementary product developed novel and superior quality. Hence in this study, the energy, protein and Fe densities of *dabi* teff-field pea-based optimised novel complementary flour and its contribution to daily energy and nutrient demand by 6–23-month-old children were investigated in comparison with FAO/WHO^(12)^ recommended nutrient contents of complementary foods and PAHO/WHO^(15)^ recommended daily energy and nutrients requirements for 6–23-month-old children to ensure adequate energy and nutrients daily intake from the optimised complementary flour for children’s optimal growth and development.

## Materials and methods

### Food crops sample collection

**Fig. 1.**
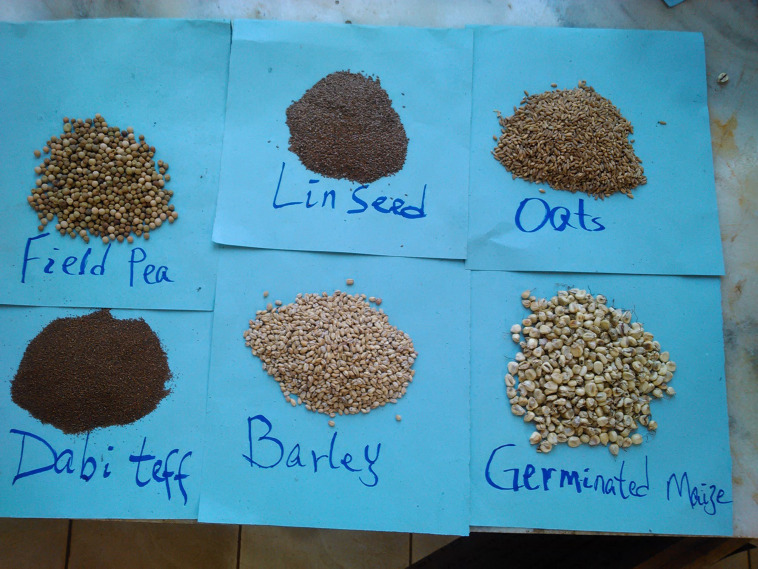
Collected complementary food crops (photo by the researcher).

Eight mothers who were judged to have good knowledge of complementary foods assisted by health extension workers in the village were asked about the types and affordability of common complementary crops used for feeding infants and young children in Nedjo area, west Wollega, Ethiopia and its presence in the market was inspected. Six food samples including *dabi* teff *(Eragrostis tef (Zucc.) farmer variety*), maize (*Zea mays L.*), barley (*Hordeum vulgare*), white field pea (*Pisum sativum*), oats *(Avena sativa)* and linseed (*Linum usitatissimum*) (Fig. 1) were purchased from an open market of Nedjo town, which is located at 575 km away to the west of Addis Ababa where Nedjo district is a potential *dabi* teff growers. The nutrient contents of these crops were initially literature searched, later verified through laboratory experimentation (nutrient analysis of each crop) (Table 1) and were carefully selected to add one or more nutrients to their mixture to be nutritionally complemented. Three kilograms of each apparently healthy sample was purchased from the centre and corners of the market on two different market days to assure representativeness, packed separately in polyethylene bags and transported to the Food Science and Nutrition Laboratory of Ethiopian Institute of Agricultural Research for analysis. All laboratory analysis of the nutritional composition of each crop sample and the formulated complementary food was conducted in the stated laboratory, which was certified by the International Organization for Standardization (ISO-17025:2017) by International Laboratory Accreditation Cooperation.

**Table 1.** Major macro- and mineral compositions of each crop sample collected

Crop samples	Macro-compositions (g/100 g)				Energy (kcal/100 g)	Dietary minerals (mg/100 g)					
	Protein	CHO	Fat	Ash		Fe	Zn	Ca	Mg	P	K
Dabi teff	10·74^a^	68·57^a^	3·94^a^	3·96^a^	352·70^a^	86·50^a^	1·94^a^	123·59^a^	142·48^a^	237·14^a^	297·97^a^
Roasted field pea	20·95^b^	60·45^b^	2·74^a^	2·60^b^	350·26^a^	7·05^b^	2·28^a^	59·99^b^	89·54^b^	227·58^a^	720·14^b^
Roasted barley	11·62^a^	77·63^c^	2·58^a^	1·73^c^	380·22^b^	5·00^b^	2·46^a^	32·49^c^	87·48^b^	279·09^b^	355·45^c^
Dehulled oats	12·45^a^	70·00^a^	5·69^a^	1·92^c^	381·01^b^	4·37^b^	2·28^a^	26·33^d^	78·87^b^	261·23^b^	319·04^c^
Cooked linseed	20·57^b^	28·24^d^	36·08^b^	3·45^a^	519·96^c^	47·13^c^	6·63^b^	28·83^d^	279·86^c^	488·97^c^	563·28^d^
Germinated maize	8·58^c^	75·24^c^	4·61^a^	1·42^c^	376·77^b^	1·12^d^	1·56^c^	15·78^e^	80·91^b^	236·48^a^	260·20^a^

Values are means of the triplicate determinations. Values in the same column followed by different superscripts are significantly different at *P* < 0·05.

### Sample processing

**Fig. 2.**
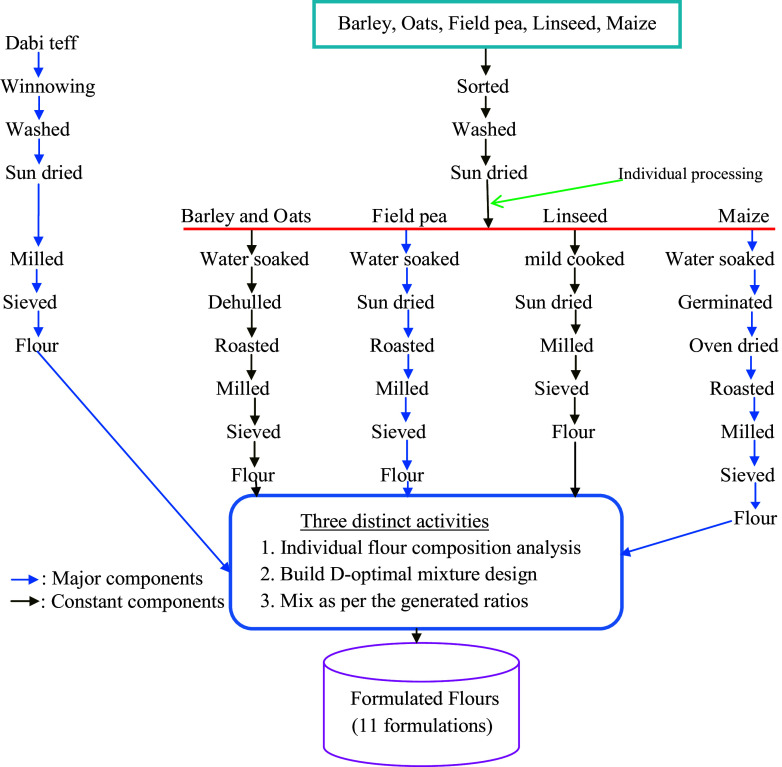
Process flow chart diagram of the crop samples and formulations.

The collected crop samples underwent various controlled processing techniques (Fig. 2). In brief, *dabi* teff was manually cleaned by winnowing to remove chaff, straw, dust and other extraneous and washed with tap water and sundried for 2 d at an average temperature of 27°C while the other cereals (maize, barley and oats) and the legumes (field pea and linseed) were sorted out from sands, sticks, stones and defective seeds, later washed and sundried for 2 d at an average temperature of 27°C and were ready for further individual processing. Due to the small size of *dabi* teff seeds, it was made into whole-seed milled flour, which could be the reason for the higher nutrient contents of the crop. Barley and oats samples were soaked in clean tap water for 2 h. The water used for soaking was drained off and the crops were immediately decorticated (while the seeds were still wet) using a wooden decorticator and the hulls were removed by winnowing.

Germination of maize seeds was adopted from the method previously described by Rasane *et al.* ^(40)^ with little modification. Briefly, the maize grains were soaked in water (1:3 w/v) for 3 h to achieve hydration, drained, spread on a clean jute sack placed on a wooden platform and covered with another jute sack for germination at room temperature (25 (sd 2)°C) for 72 h and water was sprayed every 12 h to stay humid (60 % relative humidity). This 72 h was used because Rasane *et al.* ^(40)^ reported that the maximum amylase activity was obtained at 72 h of germination time. After 72 h, the germinated seeds were rinsed, drained for 10 min, transferred to aluminium trays and dried in an air oven at 40°C for 5 h to terminate the germination process. The dried germinated sample was further roasted at 120 (sd 5)°C for 10 min and allowed to cool.

The crop samples viz., barley, oats, field pea and germinated maize were minimally roasted in an oven at 120°C for 20 min until light brown coloured and then cooled to room temperature (25 (sd 2)°C) as described by Rasane *et al.* ^(40)^. The roasting process was carefully controlled to prevent over-roasting and the formation of a Maillard reaction that may lead to protein quality damage. The linseed sample was minimally cooked for 5 min at 90°C^(41)^ with a small amount of water to condition the seeds to release oil from the oil cells and later sundried without draining the water used for cooking.

### Flour preparation and handling

All the six processed samples were milled into flours using a standard miller (Cyclotec 1093 sample mill, Foss Analytical) to obtain smooth and consistent particle sizes and sieved through a 0·5 mm mesh sieve size. The flours were then packed in air-tight high-density polyethylene bags as described by the American Association of Cereal Chemists^(42)^, separately coded and stored safely at room temperature till formulation.

### Experimental design

#### Flour formulation

A mixture of cereals, legumes, pulse and/or oilseed flours constitutes an appropriate source of nutrients and energy density, essential fatty acids and limiting amino acids and ends up with many functional and health benefits as well as improved organoleptic characteristics^(12)^. In the current study, Stat-Ease Design-Expert^®^ software 11 (Randomized Mixture Design, D-optimal, Minneapolis, USA, 2018) was used to generate the formulation design matrix.

#### Macro-compositions analysis

In this study, Associations of Official Analytical Chemists (AOAC)^(43)^ modified methods were used to determine the macro-composition of each crop sample and the formulated complementary flours. The moisture content was determined by air convection drying oven (Model No. DHG-9121A) using the method described by 925.10, AOAC^(43)^ for 1 h at 130 (sd 3)°C. Crude protein content was determined by Kjeldahl (Kjeltec 8400, Auto Sample Systems, Foss Analytical) using a nitrogen conversion factor of 6·25 following the official method 954.10, AOAC^(43)^. Soxhlet method (Soxtec 8000, Tecator Line, Foss Analytical) was used to determine the crude fat content by N-Hexane extract according to the method number 2003.06, AOAC^(43)^. Ash content was determined using the combustion method by box type muffle furnace (Model No.SX2-4-10GJ, China) at 550°C for 4 h following method 923.03, AOAC^(43)^. Crude fibre content was determined by Fibertec 8000, Foss analytical, following official method 978.10, AOAC^(43)^. Utilisable carbohydrate was estimated by difference: 100 - (% Moisture + % Crude protein + % Crude fat + % Crude fibre + % Ash)^(44)^. The gross energy (kcal/100 g) of each crop sample and the formulated flours were computed based on FAO/WHO^(45)^ recently amended in the 2021 codex guideline by multiplying the values obtained for crude protein, crude fat and utilisable carbohydrate (the energy-yielding macronutrients) with Atwater conversion factors where E (kcal/100 g) = ((% crude fat × 9) + (% crude proteins × 4) + (% utilisable carbohydrates × 4)].

#### Dietary mineral analysis

Analysis of aqueous solutions by inductively coupled plasma-optical emission spectrometry with radial plasma observation method was used to determine the dietary mineral contents (Fe, Zn, Ca, Mg, K, P, Na) of each crop sample and the formulated complementary flours with radial plasma observation method^(46)^. All the measurements were performed using a sector ARCOS optical emission spectrometer optimised with small volume and thirty-two linear charge-coupled devices detectors in the wave length range between 130 and 770 nm and simultaneously analysed. A nebuliser was used to introduce sample solution into the argon gas field plasma and the energy excitement was proceeded in the single spectra and the energy was expressed in intensity, which is directly proportion to concentration, the concentration was calculated on the linear graph of the standard concentration and the corresponding intensities. All the measuring conditions, like plasma power, gas flows, torch positions and measuring time, are configured. It was a concentration measurement. According to the standard, the calibration and standardisation of the spectra method were performed and standardisation was done daily and it is a quick procedure for correcting measuring intensities so that the correct concentration is obtained using the original calibration curve.

#### Variables optimisation and validation

Numerical and graphical optimisation techniques were employed to identify the optimum formula (sweet spot) using the Deign-Expert (D-optimal mixture design). Simultaneous numerical values of independent variables and multi-responses optimisation were performed by setting the desired goals for each independent and dependent (response) variable. In brief, the major components used; *dabi* teff, field pea and germinated maize flours were set in range and the responses were set to maximise energy, protein, ash, fat, carbohydrate, Fe, Ca and Zn; whereas to minimise the moisture and crude fibre (Table 2). In addition to goal setting, the relative importance of each dependent variable in the overall optimisation solution was set to be ‘++++’ for protein and carbohydrate while it was set as ‘+++++’ for Fe, Ca and Zn.

**Table 2.** Goals set, the relative importance of each variable and the optimal values at optimal conditions

Variables name	Goal	Lower limit	Upper limit	Importance	Optimum value
Independent variables					
A: Dabi teff flour	In range	20	35	3	34·66
D: Field pea flour	In range	0	30	3	15·34
F: Maize flour	In range	5	20	3	5
Barley flour	Constant	25	25	3	25
Oats flour	Constant	15	15	3	15
Linseed flour	Constant	5	5	3	5
Dependent variables					
Protein (%)	Maximise	14·58	17·21	4	15·74
Fat (%)	Maximise	4·22	5·59	3	5·09
Ash (%)	Maximise	2·01	2·6	3	2·26
Crude fibre (%)	Minimise	2·68	3·96	3	2·88
Carbohydrate (%)	Maximise	66·91	70·76	4	73·05
Energy (kcal/100 g)	Maximise	378·82	386·9	3	380·43
Fe (mg/100 g)	Maximise	24·007	31·5801	5	32·21
Ca (mg/100 g)	Maximise	73·4631	78·1767	5	77·51
Zn (mg/100 g)	Maximise	2·326	2·612	5	2·59

The graphical optimisation was carried out by superimposition of contour plots for all the responses with respect to component ratios. To confirm the validity of the optimisation, a laboratory experiment was conducted on the component ratios identified at the optimal formula and the obtained results were then compared with predicted values of the responses at optimal conditions and with values of the control flour and finally reported as the formula with optimal nutritional (macro-and micronutrients) profile of all the possible combinations or solutions.

#### Determination of energy and nutrient Densities of the optimised formula

The energy density of complementary foods, expressed as kcal/g, can be calculated by dividing the total energy from complementary foods by the amount in g of complementary foods served to the children per day, taking into account the theoretical gastric capacity of infants and young children to be 30 g/kg body weight per meal and the required number of meals to be served to the children per day for the respective age group. However, this theoretical gastric capacity may not usually be functional due to a lack of responsive feeding, inappropriate consistency of the foods or underlying health conditions where the children take less^(24)^.

The nutrient densities of the optimised flour were computed by dividing the respective nutrient contents obtained at optimal condition by energy value at optimal and given as g/100 kcal for macro and mg/100 kcal for micronutrients. To make it brief, the protein density or protein-energy ratio was calculated by dividing the crude protein content of the optimised flour by its corresponding energy value expressed as g/100 kcal. Similarly, the Fe density or Fe energy ratio was calculated by dividing the Fe content of the optimised flour by its corresponding energy value expressed as mg/100 kcal.

The amount of food needed per day by children in their respective age groups to satisfy their energy and nutrient intake demand is a function of the amount of energy and nutrient densities that can be provided by the complementary foods^(14)^. Abeshu *et al.* ^(23)^ estimated that for complementary food having energy density range of 0·6–1·0 kcal/g, the amount of food served to provide the daily energy requirement is 263·3 g for 6–8-month-old children, 317·09 g for 9–11-month-old children and 334·92 g for 12–23-month-old children for the study done at Wolayita Zone of Ethiopia and the children could consume an average amount of 212·82 g of the complementary food per day due to their low functional gastric capacity. Another report by Wasswa *et al.* ^(47)^ stated that the amount of porridge eaten by children in rural settings of Uganda was estimated at around 200 g per day for children aged 6–12 months.

Consequently, based on these two reports and the Ethiopian complementary feeding guidelines^(48)^, we have estimated the daily amounts of our optimised complementary food to be served to an age group of 6–8, 9–11 and 12–23-month-old children to meet their daily requirements to be 225, 300 and 450 g, respectively (i.e. 75, 100 and 150 g per meal for the respective age group) and 3 meals/d. The lower the energy density of a complementary diet, the greater the quantity of the diet required to meet the energy and nutrient demands by infants and young children^(14)^. PAHO/WHO^(15)^ and WHO^(20)^ complementary feeding guideline recommends that 5 meals/d are needed for a lower energy density of 0·6 kcal/g, 4 meals/d for 0·8 kcal/g and 3 meals/d when the energy density is at least 1·0 kcal/g. The standard also specifies that meal frequency that is greater than necessary may lead to excessive displacement of breast milk and preparing and feeding five meals per day requires a considerable amount of time and effort by caregivers. This may prompt them to hold prepared food over from one meal to the next, thereby potentially increasing the risk of microbial contamination which needs to be considered when developing messages regarding meal frequency due to which 1–2 nutritious snacks per day, such as a piece of fruit or a piece of bread would be recommended that takes less time for preparation and may also be less likely to displace breast milk^(15)^.

#### Contribution of the optimised complementary flour to the daily recommended energy and nutrient intake demand by infants and young children

Considering the estimated daily amount of complementary food to be served to the children and the amount of daily energy and nutrient demand by the respective age group as described in the introduction section above, the likely contribution (percentage) of the optimised complementary flour to the recommended daily requirement specified by PAHO/WHO^(15)^ was computed. Further, the energy and nutrient content of the optimised flour was compared with the recommended value specified by FAO/WHO^(12)^ standards, which were also described in the introduction section earlier.

### Statistical analysis

Numerical multi-response optimisation was performed using the Design-Expert^®^, D-optimal version 11 to identify the optimal condition while one-way ANOVA of SPSS version 24 was employed to test whether there is a significant difference between the nutrient contents of the optimised, the Cerifam^®^ faffa (the popular commercial complementary flour in Ethiopia) and the control flours and significant differences were declared at *P* < 0·05. The mean nutrient contents were checked against the critical limits set by FAO/WHO^(12)^. The energy density (kcal/g), protein, carbohydrate and fat densities (g/100 kcal), and Fe, Ca and Zn densities (mg/100 kcal) of the optimised flour were compared with PAHO/WHO^(15)^ recommendations and finally, the likely daily energy and nutrients contribution by the optimised complementary flour, the Cerifam^®^ faffa and the control flour were computed for 6–8, 9–11 and 12–23-month-old children after adjusting for an estimated daily amount to be served to the children and compared with the RDA as per the PAHO/WHO^(15)^.

## Results

### Macro- and mineral compositions of each crop and the formulated complementary flours

The mean macro-compositions of each crop samples collected are presented in Table 1 where field pea and linseed flours were determined to contain a significantly higher (*P* < 0·05) protein content at 20·95 and 20·57 %, respectively, while barley, oats, maize and linseed were determined to contain a significantly higher (*P* < 0·095) energy value at 380·22, 381·01, 376·77 and 519·96 kcal/100 g, respectively, which were the bases to develop protein and energy-dense complementary flour. On the other hand, *dabi* teff has shown to contain significantly higher (*P* < 0·05) Fe and Ca content at 86·50 and 123·59 mg/100 g, respectively, which was the bases to develop Fe-dense novel complementary flour. The mean macro-compositions of the formulated complementary flours that were subjected to the optimisation process were 4·89%, 15·49, 4·91, 2·23, 3·48, 68·99 % and 382·14 kcal/100 g for moisture contents, protein, fat, ash, fibre, carbohydrate and energy contents, respectively, whereas the mean dietary mineral contents of the formulated complementary flours that were subjected to the optimisation process were 28·12, 76·25, 2·47, 120·57, 287·99 and 439·33 mg/100 g for Fe, Ca, Zn, Mg, P and K, respectively (Table 3).

**Table 3.** Macro- and mineral composition of the formulated mean, the optimised, the control and FAO/WHO specifications

Complementary flours	Macro-compositions (g/100 g)					Energy (kcal/100 g)	Dietary minerals (mg/100 g)					
	Protein	CHO		Fat	Ash		Fe	Zn	Ca	Mg	P	K
Mean of the formulated	15·49^a^	68·99^a^		4·91^a^	2·23^a^	382·14^a^	28·12^a^	2·47^a^	76·25^a^	120·57^a^	293·25^a^	443·99^a^
Optimised flour	15·74^a^	73·05^b^		5·09^a^	2·26^a^	380·43^a^	32·21^a^	2·59^a^	77·52^a^	118·64^a^	287·99^a^	439·33^a^
Control flour	10·77^b^	74·41^b^		3·78^b^	2·24^a^	374·74^b^	7·25^b^	2·64^a^	49·45^b^	96·45^b^	282·65^a^	398·67^b^
Cerifam® faffa flour	14^c^	76c		4^a^	2·5^a^	396^c^	6^c^	5^b^	100^c^	–	–	–
FAO/WHO value	≥ 15	64	4	10–25	< 3	400–425	16	3·2	500	–	–	–

Values are means of the triplicate determinations. Values in the same column followed by different superscripts are significantly different at *P* < 0·05.

### Macro- and mineral compositions of the optimised complementary flours

**Fig. 3.**
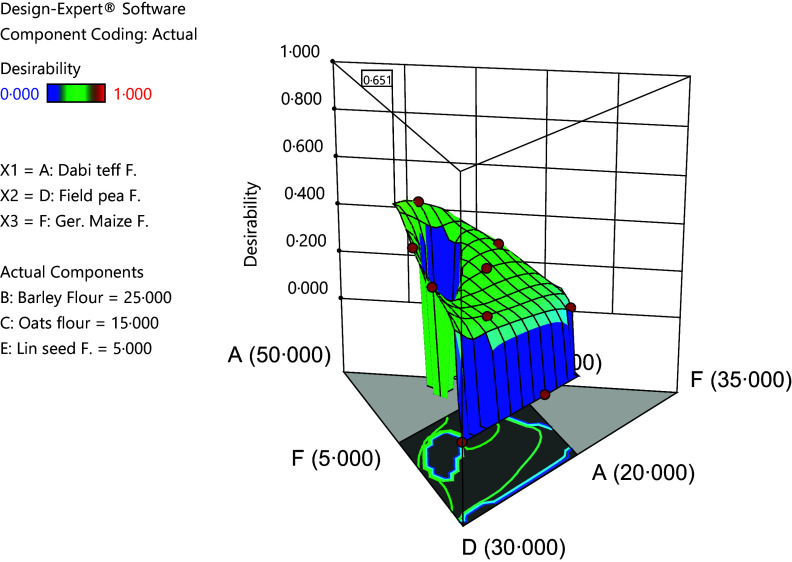
3D surface graph of desirability function at the overall optimisation.

The macro- and mineral compositions of the optimised novel complementary flour, the formulated mean, the control and the FAO/WHO^(12)^ critical limits are presented in Table 3 where the overall optimised formula was identified to be 34·66 % *dabi* teff, 25 % barley, 15 % oats, 15·34 % field pea, 5 % linseed and 5 % germinated maize flours with response values of 15·74 % protein, 73·05 % carbohydrate, 5·09 % fat and 380·43 kcal/100 g energy, 32·21 mg/100 g Fe, 77·52 mg/100 g Ca and 2·59 mg/100 g Zn, 118·64 mg/100 g Mg, 287·99 P and 439·33 mg/100 g K at the optimal point with the overall desirability function of 0·651 (Fig. 3). The control flour contained 10·77 %, 74·41 %, 3·78 % and 374·74 kcal/100 g for protein, carbohydrate, fat and energy contents, respectively, whereas it had 7·25 mg/100 g Fe, 49·45 mg/100 g Ca, 2·64 mg/100 g Zn, 96·45 mg/100 g Mg, 282·65 mg/100 g P and 398·67 mg/100 g K, respectively. Cerifam^®^ faffa flour contains 14 % protein, 76 % carbohydrate, 4 % fat and 396 kcal/100 g, and it contains 6 mg/100 g Fe, 5 mg/100 g Zn and 100 mg/100 g Ca, respectively (Table 3). Both the formulated mean and the optimised value fulfilled the FAO/WHO^(12)^ critical standard limit. The energy, protein and Fe contents of the optimised and the formulated mean were significantly higher (*P* < 0·05) than the values for the control and the Cerifam^®^ faffa flours, while there was no significant difference (*P* > 0·05) between the ash and Zn contents of the optimised, the formulated mean, the control and the Cerifam^®^ faffa flours.

**Fig. 4.**
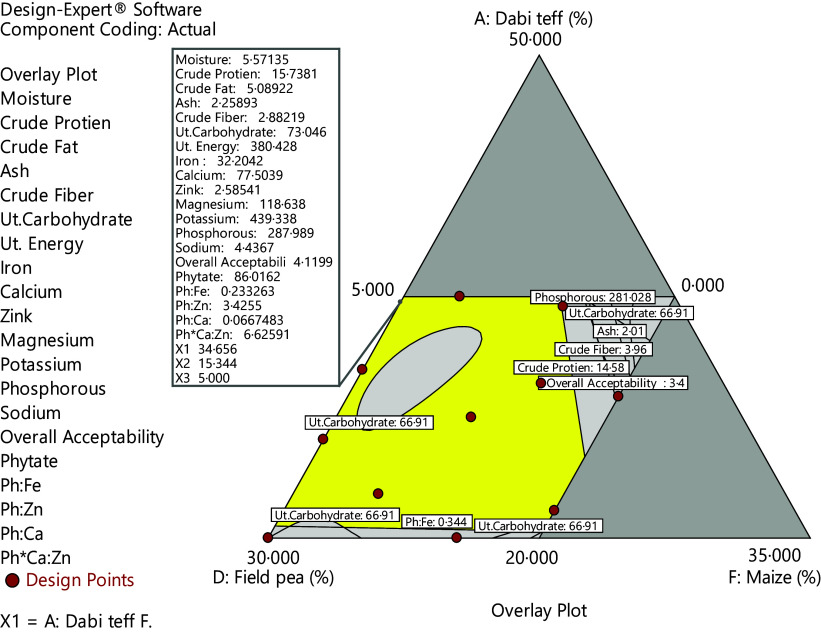
Graphical overlay contour plot for the overall graphical optimisation.

The validation analysis result was 5·34 %, 15·86 %, 5·12 % 2·21 %, 2·85 %, 68·82 %, 384·80 kcal/100 g, 31·76 mg/100 g, 76·88 mg/100 g and 2·53 mg/100 g for moisture, protein, fat, ash, fibre, energy, Fe, Ca and Zn contents, respectively (Table 4). This showed that there was a good agreement between the predicted response values at optimal conditions and the validation laboratory values. The optimisation process was verified using the graphical optimisation technique (Fig. 4) where the overlay counter plot was generated for the most important variables to identify the optimum regions. Any design points that fall within the yellow region (with the very conservative silver-grey region) in the overlay plot represent an optimal combination of *dabi* teff flour, field pea and germinated maize flours in combination with the other constant components that can bear optimal macro- and micronutrient contents within 95 % CI.

**Table 4.** Experimental validation of optimal condition and comparison with the control flour

Response variables	Optimal value	Validation value	Control value
Protien (g/100 g)	15·74^a^	15·86^a^	10·77^b^
Fat (g/100 g)	5·09^a^	5·12^a^	3·78^b^
Ash (g/100 g)	2·26^a^	2·21^a^	2·24^a^
Fibre (g/100 g)	2·88^a^	2·85^a^	2·65^a^
Carbohydrtae (g/100 g)	73·05^a^	68·82^a^	74·41^a^
Energy (kcal/100 g)	380·43^a^	384·80^a^	374·74^b^
Fe (mg/100 g)	32·21a	31·76a	7·25b
Ca (mg/100 g)	77·51^a^	76·88^a^	49·45^b^
Zn (mg/100 g)	2·59^a^	2·53^a^	2·64^a^

Values are means of the triplicate determinations. Values in the same column followed by different superscripts are significantly different at *P* < 0·05.

### Energy and macro-nutrient densities of the optimised, the control flours and PAHO/WHO recommendation

The energy and macro-nutrient densities of the formulated mean, the optimised, the control flours and the PAHO/WHO^(15)^ recommendations for complementary food for average breastfed children are presented in Table 5. After adjusting for the amount of food to be served to the children, the energy density of our current optimised complementary flour for all the age groups (6–8, 9–11 and 12–23-month-old children) was computed to be 1·27 kcal/g. The protein density of our optimised complementary flour was computed to be 4·14 g/100 kcal and which was 1·44 times higher than the protein density of the control (traditional) flour while the fat density was computed to be 1·34 g/100 kcal and the carbohydrate density of the optimised flour was computed to be 19·20 g/100 kcal.

**Table 5.** Energy and nutrient densities of the formulated mean, the optimal and the control flours and PAHO/WHO standards

Parameters	Formulated mean	Optimal	Control	PAHO/WHO
Energy density (kcal/g)	1·27	1·27	1·25	≥ 0·8
Protein density (g/100 kcal)	4·05	4·14	2·87	≤ 5·5
Fat density (g/100 kcal)	1·55	1·34	1·01	2·5–4·5
Carbohydrate density (g/100 kcal)	18·05	19·20	19·86	–
Fe density (mg/100 kcal)	7·36	8·47	1·93	0·8–4
Ca density (mg/100 kcal)	19·95	20·37	13·20	>80
Zn density (mg/100 kcal)	0·65	0·68	0·70	0·6–1·6

Source of PAHO/WHO: Codex Stan 074–1981, Rev. 1–2006; PHO/WHO, 2001.

### Minerals densities of the optimised, the control flours and PAHO/WHO recommendation

The dietary minerals densities of the formulated mean, the optimised and the control flours and the PAHO/WHO^(15)^ recommendations for complementary food for average breastfed children are presented in Table 5. The Fe density of our optimised complementary flour was computed to be 8·47 mg/100 kcal, and it was 4·39 times higher than the control which could be accredited to the higher Fe content of *dabi* teff flour at 86·50 mg/100 g (Table 1) which was one of the major ingredients in our formulations. The Zn density of our optimised flour was determined to be 0·68 mg/100 kcal and the Ca density was determined to be 20·37 mg/100 kcal.

### Contribution of the optimised complementary flour

#### Contribution of the optimised complementary flour to daily energy and protein demand by 6–23-month- old children

Table 6 shows the required daily meal frequency and the estimated amount of solid portions of complementary food per meal for age groups of 6–8, 9–11 and 12–23-month-old children and the recommended daily requirements of energy and protein from a complementary food based on PAHO/WHO Guiding *Principles for Complementary Feeding of the Breastfed Child.* After adjusting for the amount of complementary food a child in its respective age group needs to be served, the likely daily contribution (in percentage) of the currently developed optimal complementary food to the recommendation and the daily requirements was computed for energy and protein. It was further compared with the relative contribution of the control and Cerifam^®^ faff flour (the popular commercial complementary flour in Ethiopia). In the above section, it was estimated that the daily amounts of complementary foods to be served to an age group of 6–8, 9–11 and 12–23-month-old children to be 225, 300 and 450 g, respectively. Based on adjusting for this estimated daily serving amount and the daily requirements of the nutrients, the currently developed optimised complementary product could likely contribute up to 143, 127 and 104 % of daily energy requirements for the age group of 6–8, 9–11 and 12–23-month-old child, respectively (Table 6). Similarly, after adjusting, the optimised complementary flour could be able to contribute up to 130, 164 and 217 % of the daily protein requirements for the respective age groups. This contribution of the optimised complementary flour to the PHO/WHO recommended requirements (demand) was significant (more than 100 %) and the product can considerably fulfil the energy and protein demand by the children under each respective age group, which showed the protein-energy deficit would be less likely to occur if the children would be fed the estimated serving amount properly.

**Table 6.** Contribution of the optimised flour to the daily energy and protein demands by 6–23-month-old children from complementary flour as recommended by PAHO/WHO (2001)

Nutrient intake	Age group					
	6–8 months		9–11 months		12–23 months	
Servings						
Number of servings (meal)	3 times		3 times		3 times	
Amount of solid portion per meal (g)	75		100		150	
Energy RDA from CF (kcal/d)	200		300		550	
Flour type	Adjusted	% CTB	Determined	%CTB	Adjusted	% CTB
Optimised formula	285·32	142·66	380·43	126·81	570·65	103·75
Control	281·06	140·53	374·74	124·91	562·11	102·20
Cerifam^®^ faffa	297	148·50	396	132	594	108
Protein RDA from CF (g/d)	9·1		9·6		10·9	
Flour type	Adjusted	% CTB	Determined	%CTB	Adjusted	% CTB
Optimised formula	11·81	129·78	15·74	163·96	23·61	216·61
Control	8·08	88·76	10·77	112·19	16·16	148·53
Cerifam^®^ faffa	10·5	115·38	14·0	145·83	21·0	192·66

PAHO/WHO, 2001; CF, complementary food; %CTB, percentage contribution.

The relative contributions of the optimal, the control and the Cerifam^®^ faffa flour to the daily energy and protein demand by the children are presented in Table 6, which was calculated to be 143, 141 and 149 % by the optimal, the control and Cerifam^®^ faffa flours for the 6–8-month-old children, 127, 125 and 132 % for the 9–11-month-old children and 104, 102 and 108 % for the age group of 12–23-month-old children, respectively. When compared with the control and the Cerifam^®^ faffa flour, the contribution of energy by the optimised complementary flour was slightly higher than that of the control flour and slightly lower than that of the Cerifam^®^ faffa flour for the entire age category. This relatively comparable energy content of the control and Cerifam^®^ faffa flour to the optimal flour could be attributed to the cereals, barley and wheat flours in their formulation, which are claimed as higher energy-containing cereals. The relative contribution of the optimal complementary flour to the daily protein demand was calculated to be higher than both the control and Cerifam^®^ faffa flour and was determined to be 130, 89 and 115 % by the optimal, control and Cerifam^®^ faffa flours for the 6–8-month-old- children, 164, 146 and 112 % for the 9–11-month-old children and 217, 148 and 193 % for the age group of 12–23-month-old children, respectively (Table 6).

#### Contribution of the optimised complementary flour to daily Fe, Ca and Zn demand by 6–23-month-old children

Table 7 shows the required daily meal frequency and the estimated amount of solid portions of a complementary food per meal for age groups of 6–8, 9–11 and 12–23-month-old children and the recommended daily requirements (RDA) of Fe, Ca and Zn from a complementary food based on PAHO/WHO Guiding *Principles for Complementary Feeding of the Breastfed Child.* After adjusting for the estimated daily serving amount and the daily requirements of the minerals as well as based on the assumption of moderate bioavailability, the currently optimised complementary flour was computed to contribute up to 220, 293 and 690 % of the daily Fe requirement for 6–8, 9–11 and 12–23-month-old children, respectively, with daily Fe demand of 11, 11 and 7 mg by 6–8, 9–11 and 12–23-month-old children, respectively (Table 7). This contribution of the optimised complementary flour to the PHO/WHO recommended requirements (demand) for Fe was significant (more than 200 %) and the product can considerably fulfil the Fe demand by the children under each respective age group, which showed the Fe deficiency anaemia would be less likely to occur if the children would be fed the estimated serving amount properly.

**Table 7.** Contribution of the optimised flour to the daily Fe, Ca and Zn demands by 6–23-month-old children from complementary flour as recommended by PAHO/WHO (2001)

Nutrient intake	Age group					
	6–8 months		9–11 months		12–23 months	
Servings						
Number of servings (meal)	3 times		3 times		3 times	
Amount of solid portion per meal(g)	75		100		150	
Fe RDA from CF (mg/d)	11		11		7	
Flour type	Adjusted	%CTB	Determined	%CTB	Adjusted	%CTB
Optimised formula	24·16	219·61	32·21	292·82	48·32	690·29
Control	5·44	49·45	7·25	65·91	10·86	155·14
From Cerifam^®^ fafa	4·5	40·91	6	54·55	9	128·57
Zn RDA from CF (mg/d)	4·1		4·3		5·8	
Flour type	Adjusted	%CTB	Determined	%CTB	Adjusted	%CTB
Optimised formula	1·94	47·32	2·59	60·23	3·89 (67)	67·07
Control	1·98	48·29	2·64	61·39	3·96	68·28
Cerifam^®^ faffa	3·75	91·46	5	116·28	7·5	129·31
Ca RDA from CF (mg/d)	400		400		500	
Flour type	Adjusted	%CTB	Determined	%CTB	Adjusted	%CTB
Optimised formula	58·13	14·53	77·5	19·37	116·25	23·25
Control flour	37·09	9·27	49·45	12·36	74·18	14·84
Cerifam^®^ faffa	75	18·75	100	25	150	30

PAHO/WHO, 2001; CF, complementary food; %CTB, percentage contribution. All the calculations were based on the moderate bioavailability of the minerals.

Regarding Zn, the optimised flour was demonstrated to contribute up to 47·32, 60·23and 67·07 % of daily Zn demand of 4·1, 4·3 and 5·8 mg by the respective age groups. On the other hand, the new optimised *dabi* teff-field-based complementary flour could only contribute up to 14·53, 19·37 and 23·3 % of daily Ca requirement (demand) of 400, 400 and 500 mg by the respective age group (Table 7).

Regarding the relative Fe contribution by the optimised complementary flour to the daily Fe demand by the children, the optimal flour had a significantly higher (non-comparably higher) percentage contribution than both the control and Cerifam^®^ faffa flour where it was calculated to be 220, 50 and 41 % by the optimal, the control and the Cerifam^®^ faffa flours for the 6–8-month-old children, 293, 66 and 55 % for the 9–11-month-old children and 690, 155 and 129 % for the age group of 12–23-month-old children, respectively (Table 7). This higher Fe contribution by the optimised complementary flour was ascribed to the higher Fe content of *dabi* teff flour at 86·5 mg/100 g (Table 1).

On the other hand, the relative contribution by the optimised complementary flour to daily Zn demand was comparable with that of the control flour, while it was lower than that of the Cerifam^®^ faffa flour for all the age groups where it was calculated to be 47, 48 and 91 % by the optimal, the control and the Cerifam^®^ faffa flours for the 6–8-month-old children, 60, 61 and 166 % for the 9–11-month-old children and 67, 68 and 129 % for the age group of 12–23-month-old children, respectively (Table 7) and this higher Zn content of the Cerifam^®^ faffa flour could be attributed to the ingredients used during its formulation. Regarding the relative contribution of the three flours to daily Ca demand, the optimised, the control and the Cerifam^®^ faffa flour were found to be very low for all the age groups ranging from 9 % to 30 % (Table 7) with relatively higher contribution by Cerifam^®^ faffa flour, which could account for the milk powder in its formulation.

## Discussion

The complementary crop samples collected have shown to contain varied and higher energy and nutrient contents, and each of the crop samples was designed to add one or more nutrients to their mixture, which were used to develop nutritionally complemented optimised novel complementary flour.

When compared with the FAO/WHO^(12)^ recommended guideline for nutrient content requirements of formulated complementary foods, the mean macronutrients values of the formulations and the response values at the optimal condition of the currently developed complementary flour were adequately met the recommended critical limits for all the macro-compositions except for fat content at ≤ 5, ≥ 15, 10–25, ≤ 3, ≤ 5, 64 (sd 4) % and 400–425 kcal/100 g for moisture, protein, fat, ash, fibre, carbohydrate and energy contents, respectively (Table 3). Similarly, the mineral contents adequately met the recommended critical limits for Fe at 16 mg/100 gm for both the formulated mean and the optimised complementary flours (Table 3) where the mean formulation was 1·76 times higher and the optimal value was 2·01 times higher than the recommendation for Fe content. The Ca and Zn contents, however, did not meet the recommendation at 500 and 3·2 mg/100 g, respectively, though Zn could meet up to 77·19 % of the required value, which was marginal.

According to PAHO/WHO^(15)^ complementary feeding guidelines, the energy density of cereal-based complementary foods for infants and young children should be ≥ 0·8 kcal/g. Hence, our optimised complementary flour had demonstrated to meet beyond the minimum energy density requirements from complementary foods at 1·27 kcal/g for infants and young children aged 6–23 months old and the children can meet their daily energy demand if served the estimated amounts for the respective age group described in the above section and if fed three times per day as the guideline specifies that 3 meals/d would satisfy the energy demand of the children if the complementary food contains an energy density of at least 1 kcal/g.

It was unable to compare the energy density of our current optimised product with some past reports because of the wrong energy density calculation which said, ‘Simply dividing the energy contents of the complementary foods by 100’. For example, Tenagashaw *et al.* ^(49)^ reported energy density of complementary food formulated from blends of teff, soyabean and orange-fleshed sweet potato ranged from 3·70 to 3·76 kcal/g, and Ayele *et al.* ^(44)^ reported the energy density of complementary foods from kocho, pumpkin fruit, red kidney bean and maize at 3·3 kcal/g following the same calculation, which was overexaggerated. Even higher energy-dense foods have energy density of 1·07–1·46 kcal/g where in this case, the approximate quantity of complementary food that would meet the energy needs of the children is 137–187 g/d at 6–8 months, 206–281 g/d at 9–11 months and 378–515 g/d at 12–23 months old as specified by PAHO/WHO^(15,23)^ recommendation. However, the current energy density determined was higher than the energy density reported by Abebe *et al.* ^(11)^ where the traditional corn-based porridge consumed as complementary foods in rural villages of Sidama zone of Southern Ethiopia contained an energy density of 0·53 kcal/g, while kocho-based contained 0·49 kcal/g, both of which were very low energy density levels.

The FAO/WHO Codex Alimentarius committee recommends the protein density of cereal-based formulated complementary foods for infants and young children should not exceed 5·5 g/100 kcal (upper threshold) where the determined protein density of our optimised complementary flour at 4·14 g/100 kcal was near to the upper threshold. This showed that our optimised complementary food is super and can meet the recommended standard and could provide the daily protein demand by 6–23-month-old children if served the estimated amount in the above section for the respective age group and fed three times per day. This finding was in agreement with the report by Tenagashaw *et al.* ^(49)^ where the protein density of complementary foods from blends of teff, soyabean and orange-fleshed sweet potato was in the range of 3·50–4·79 g/100 kcal. It also agreed with the report by Ayele *et al.* ^(44)^ where the protein density of an optimised formula from kocho, pumpkin fruit, red kidney bean and maize was 3·0 g/100 kcal. Alternatively, the protein density of homemade complementary foods in Wolayita zone of Ethiopia contained lower protein density ranging from 2·13 to 2·48 g/100 kcal as reported by Abeshu *et al.* ^(11)^.

Fat is among the major factors determining the energy density of foods; it provides essential fatty acids needed for the effective absorption of fat-soluble vitamins, and it boosts the sensory qualities of processed foods, PAHO/WHO^(15)^. The fat density determined at 1·34 g/100 kcal in our optimised flour was below the recommended standard set by codex (2·5–4·5 g/100 kcal) for cereal-based complementary foods for infants and young children, FAO/WHO^(12)^. This finding was in agreement with the report by Tenagashaw *et al.* ^(49)^ that the fat density of complementary foods from blends of teff, soyabean and orange-fleshed sweet potato was in the range of 1·26–1·66 g/100 kcal where it did not even meet the minimum standard value that calls for additional fat-energy source. The current carbohydrate density of the optimised flour determined at 19·20 g/100 kcal agreed with the report by Tenagashaw *et al.* ^(49)^ that the carbohydrate density ranged from 18·22 to 20·05 g/100 kcal and this higher carbohydrate density could be attributed to the cereal–legume blends used to optimise the complementary flour, which were claimed to contain higher carbohydrate contents and maybe also due to the pre-processing techniques applied.

Dietary minerals such as Fe, Ca and Zn are the limiting nutrients in the diets of infants and young children^(24)^. The Fe density of our optimised flour as compared with the recommendation by PAHO/WHO^(15)^ complementary feeding guideline of cereal–legume-based complementary foods for infants and young children (6–24 months) accounted for high, moderate and low bioavailability of Fe where the recommended value is 2·5, 1·5 and 0·5 mg/100 kcal for 6–8, 9–11 and 12–23-month-old children when accounted for high bioavailability, while it is 4·0, 2·4 and 0·8 mg/100 kcal when accounted for moderate bioavailability and 7·7, 4·6 and 1·6 mg/100 kcal when accounted for low bioavailability for 6–8, 9–11, and 12–23-month-old children, respectively^(7,15,16)^.

The Fe density of our current optimised complementary flour that was computed to be 8·47 mg/100 kcal was 2·12–10·59 times higher than the recommended value when accounted for moderate bioavailability. This finding showed that the optimised complementary flour could even meet the recommendation when accounted for the low bioavailability of Fe density at 7·7 mg/100 kcal. Hence, it had demonstrated to meet the Fe density requirements from the complementary foods for infants and young children aged 6–23 months and the children can meet their daily Fe demand if properly fed. Such products with higher Fe content can be considered as meat substitutes given the Fe bioavailability would be comparable with that of red meat and the ‘novelty’ of this study would be accredited to the incorporation of *dabi* teff flour into the formulations containing high Fe at 86·50 mg/100 g (Table 1) and the presence of linseed which is a leading source of *α*-linolenic acid and *n*-3 PUFA that would make the formulated and optimised product super.

The present finding was higher than the Fe density of complementary blends of teff, soyabean and orange-fleshed sweet potato that range from 2·42 to 5·19 mg/100 kcal as reported by Tenagashaw *et al.* ^(49)^. Fe in plant foods exists in non-hem form, and it may also bind with anti-nutrients found in cereal–legume foods where its absorption can be impaired^(50)^; but in our case, the pre-processing technique applied could reduce the effect of the anti-nutrients in inhibiting absorption.

Zn is an important mineral used for the management of diseases such as diarrhoea, pneumonia and sometimes malaria in children. It is also used for tissue growth and maintenance and wound healing where its deficiency leads to stunted growth though its effect is less recognised and referred to us the ‘hidden hunger’^(14)^. PAHO/WHO^(15)^ recommends Zn density at 1·6, 1·1 and 0·6 mg/100 g for 6–8, 9–11 and 12–23 months of age children from complementary food when accounted for lower bioavailability^(7)^. In the present study, the determined Zn density at 0·68 mg/100 kcal has shown fulfilment for 12–23-month-old children while at a marginal level (61·82 %) when accounted for the requirements for 9–11-month-old children and below the recommended for 6–8-month-old children. The present finding was higher than the Zn density of complementary food at 0·4 mg/100 kcal as reported by Ayele *et al.* ^(44)^ while it was lower than the report by Tenagashaw *et al.* ^(49)^ with a Zn density range of 1·41–1·49 mg/100 kcal.

The Ca density of our optimised flour determined at 20·37 mg/100 kcal could only meet 25·46 % of the required Ca density in formulated complementary food for infant and young children as set by codex standard (> 80 mg/100 kcal) FAO/WHO^(12)^. The present finding was slightly lower than the report by Ayele *et al.* ^(44)^ which was 24·1 mg/100 kcal but by far lower than the report by Tenagashaw *et al.* ^(49)^ with a Ca density range of 60·68–67·84 mg/100 kcal from complementary blends of teff, soyabean and orange-fleshed sweet potato, which could be attributed to the variety of soyabean and the orange-fleshed sweet potato used.

Collectively speaking, our optimised complementary food was super Fe, protein and energy sources meeting the FAO/WHO^(12)^ and PAH/WHO^(15)^ recommended standard and could provide the daily energy, protein, carbohydrate, Fe and Zn (to a considerable extent) demands by 6–23-month-old children if served 225 g of the optimised complementary food for 6–8 month-old children, 300 g for 9–11-month-old children and 450 g for 12–23-month-old children and feed three times per day accompanied by additional nutritional snacks during any signs of hunger and satiety and average breast-feeding.

Of the examined nutrients, the determined fat density at 1·34 g/100 kcal and Ca density at 20·37 mg/100 kcal (Table 5) were found to be unfavourably lower than the required standard at 2·5–4·5 g/100 kcal of fat density and at > 80 mg/100 kcal of Ca density where these two nutrients were frequently reported to be lower in cereal-based complementary foods^(26,49,51)^. The lower fat and Ca densities might be matched by advising mothers/caretakers to add Ca and vitamin-rich foods such as eggs, dried meat powder (quanta powder), refined butter and vegetable oil (the usual practice), mashed vegetables and replacing water with milk as well as adding mashed vegetables during porridge preparation for infants and young children which might aid to enhance energy density and to meet the recommendations. The energy density of a meal can be increased from 0·6 kcal/g to 1·0 kcal/g by adding one teaspoon of oil/butter or one piled teaspoon of mashed avocado or some amount of meat to 100 g of porridge^(52)^.

PAHO/WHO^(15,24)^ recommends that the energy needs from complementary foods for infants and young children with average breast milk intake in developing countries are approximately 200, 300 and 550 kcal per day for 6–8 months, 9–11 months and, at 12–23 months of age children, respectively. Complementary foods are expected to be energy-dense and fill these deficits of daily energy and nutrient demand by the children apart from the amount obtained from breast-feeding^(25)^. Among the energy-yielding macronutrients, protein should provide 6–15 % of total daily energy requirements, which corresponds to 9·1, 9·6 and 10·9 g of protein per day for 6–8, 9–11 and 12–23-month-old children, respectively, PAHO/WHO^(15)^.

From the other energy-yielding macronutrients, the standard states 55–65 % of total daily energy requirements should come from carbohydrate and the suggested amount ranging from 30 to 45 % need to come from fat energy^(24)^. From the optimal points of the optimised complementary flour, the contribution of energy from protein and carbohydrate to the total energy of the complementary flour was 16·55 and 76·81 %, respectively, which was adequately considerable and agreed with the recommended values, whereas the energy from fat was determined to be 12·04 %, which was below the desired range.

From these findings, it could be deduced that the contribution of Fe that can be obtained from the optimised *dabi* teff-field pea-based complementary flour to the daily Fe demand by the children was extraordinarily high that it could even meet the daily requirement under low bioavailability. Thus, the product could demonstrate to fill the huge gap of Fe demand by children that should come from complementary food (97 %) and can serve as a sustainable food-based nutrition intervention against Fe deficiency anaemia.

It might not be surprising that the optimised complementary flour could demonstrate to contribute a higher amount of Fe because *dabi* teff flour had Fe content at 86·5 mg/100 g, which was one of the major ingredients in our formulations. It is unlikely that the high levels of Fe in the optimal complementary flour can cause any danger to infants and young children since Fe overload in body fluid is mostly hereditary where the intestine continues to absorb Fe at a high rate despite the excess Fe builds up in the body tissue. Fe deficiency is the most common problem rather than its toxicity and some adverse health effects of Fe overload like fatigue, mental depression, abnormal heartbeat, intestinal side effects and diabetes are severe with alcohol abusers because alcohol damages the intestine and impairs its defense against absorbing too much Fe^(53)^. However, the use of such high Fe-containing food in malaria-endemic areas needs to be stressed to avoid complications and may require following the WHO/UNICEF recommendation^(54)^.

The contribution of Zn by the currently optimised complementary flour was lower as compared with the report by^(55)^ for the formulation of complementary food from chickpea (*kabuli variety*), red teff (*DZ-0199 variety*) and quality protein maize (*BHQPY-545 variety and Ipomoea batatas, Tulla variety*) that the Zn content fulfilled 81–88·34 % of the RDA, which could be attributed to variety and component variation. In agreement with the present findings, Adetola *et al.* ^(56)^ reported that composite flour from orange-fleshed sweet potato, soyabean and carrot met 22–23 % of the daily recommended allowance for Ca for the 1–3 years age group.

The practical applications of the current study to the reality of shifting the optimised product into children’s diet are that *dabi* teff-field pea-based energy, protein and Fe-dense homemade complementary foods targeting infants and young children and school-going children can be prepared at home and can also be scaled up at industrial baby food production. The higher Fe density and the notably higher contribution of the product to the daily Fe requirements could make the product have paramount implications in sports nutrition. For example, recent studies have shown that athletic runners require 30–70 % more Fe due to losses from foot strike haemolysis and gastrointestinal blood loss^(57)^ where the consumption of our new product could increase the Hb level of the blood helping more oxygen to be transmitted, which might aid in the higher resistance and increase endurance during athletics performance. It is also suggested that this study needs to be substantiated by conducting randomised controlled feeding trials combined with nutrition education through behavioural change communication to mothers/caretakers to adopt, practise and maintain our new food formula preparation in the rural community and determine the efficacy of the newly developed *dabi* teff-field pea-based energy, protein and Fe-dense optimised novel complementary flour in improving the nutritional and Fe status of infants and young children.

### Conclusion

Nutritional well-being in early childhood is a major driver for optimal growth and development, healthy life, improved school performance as well as improved future productivity and earning potential of children. Children should be nourished with age-specific daily adequate energy and nutrient demands. In the present study, the optimised *dabi* teff-field pea-based novel complementary flour was demonstrated to provide adequate energy, protein and Fe densities meeting the standard and show the likely contribution of more than 100 % of daily demands for energy and protein, and notably more than 200 % of daily Fe demand by all the age groups (6–8, 9–11 and 12–23-month-old children) as per the by PAHO/WHO recommendation at moderate bioavailability where it even met at lower Fe bioavailability. Thus, children aged 6–23 months can meet their daily energy and nutrient demand if served the estimated amount of the novel complementary food and fed there times a day. The density and daily contribution of Zn from the developed product were marginal, whereas the fat and Ca densities and their daily contribution by the product were inadequate, which might require matching. This study gives the basis for the optimisation of cereal–legume blends from locally available, affordable and underutilised food crops that can serve as sustainable food-based nutrition intervention strategies to fight against protein-energy malnutrition and Fe deficiency anaemia in children.
